# Pesticides and Environmental Contaminants in Organic Honeys According to Their Different Productive Areas toward Food Safety Protection

**DOI:** 10.3390/foods9121863

**Published:** 2020-12-14

**Authors:** Sara Panseri, Elisabetta Bonerba, Maria Nobile, Federica Di Cesare, Giacomo Mosconi, Francisco Cecati, Francesco Arioli, Giuseppina Tantillo, Luca Chiesa

**Affiliations:** 1 Department of Health, Animal Science and Food Safety, Università degli Studi di Milano, Via Celoria 10, 20133 Milan, Italy; sara.panseri@unimi.it (S.P.); maria.nobile1@unimi.it (M.N.); giacomo.mosconi@unimi.it (G.M.); francesco.arioli@unimi.it (F.A.); luca.chiesa@unimi.it (L.C.); 2 Department of Veterinary Medicine, University of Bari Aldo Moro, Strada P.le per Casamassima Km3, 70010 Valenzano, Italy; elisabetta.bonerba@uniba.it; 3 INTEQUI-CONICET, Faculty of Chemistry, Biochemistry and Pharmacy, National University of San Luis, Almirante Brown 1455, San Luis 5700, CP, Argentina; fmcecati@gmail.com; 4 Department Interdisciplinary of Medicine, University of Bari, Piazza Giulio Cesare 11, 70124 Bari, Italy; giuseppina.tantillo@uniba.it

**Keywords:** pesticides, persistent organic pollutants (POPs), glyphosate, AMPA, organic honey, GC-MS/MS, IC-HRMS, food safety

## Abstract

Monitoring contaminant residues in honey helps to avoid risks to human health, as it is a natural product widely consumed in all population groups, including the most vulnerable, such as children and the elderly. This is important for organic honey production that may be negatively influenced by geographical area pollution. Considering the importance of collecting data on the occurrence of various xenobiotics in different geographical areas, this study aimed to investigate the presence of contaminant residues (persistent organic pollutants (POPs) and pesticides, including glyphosate and metabolites) in organic honey samples from different production areas using different analytical methods, in order to confirm their incidence and possible impact on the food safety traits of organic production. Regarding POPs, traces of benzofluoroanthene and chrysene were detected in honey from intensive orchards and arable lands. Traces of all polychlorobiphenyl (PCB) congeners were detected at different percentages in almost all of the samples, regardless of the origin area. Traces of polybromodiphenylethers (PBDE 28, 33, and 47) were found in different percentages of samples from all of the geographical areas examined. Traces of organochlorines (OCs) and organophosphates (OPs) were identified in honey samples belonging to all of the geographical areas. No glyphosate, glufosinate, and aminomethylphosphonic acid (AMPA) residues were detected.

## 1. Introduction

Honey bees play a key role in the environmental ecosystem as pollinating species also contributing to the production of marketed honey, beeswax, and other bee products [1]. Food safety is essential for protecting consumer health and promoting food surveillance. It is fundamental to monitor contaminant residues present in foodstuff, such as honey, to prevent health risks in humans, as it is an aliment broadly consumed throughout the population, including the most vulnerable groups, such as children and the elderly [2]. Beebread, beeswax, and honey contamination by pesticides can also affect the colonies’ vitality when contaminated matrices are present during larvae development, leading to serious ecotoxicological issues [3,4]. Moreover, honey is widely used to control oxidative deterioration processes in fruit and vegetables and/or reactions of lipid oxidation in meat [5], avoiding pathogen and microorganism proliferation that leads to the decomposition of food [6].

In modern apiculture, honey contamination may occur directly (i.e., honey bee colonies treated for veterinary purposes) or indirectly, since honey bees, during foraging, are able to cover long distances, coming in contact with polluted pollen, nectar, and water [7,8,9]. Many researchers have conducted studies on honey bees and/or honey-bee products to assess the environmental pollution level of industrial areas [1]. In fact, other works have underlined that honey contamination is strongly related to the environmental scenario considering the different types of contamination sources [2,10,11]: Pesticides applied in agriculture may consequently contaminate honey and bee products, compromising food safety [12,13]. For these reasons, in the last decades, beekeeping practices have been implemented in order to ensure human health safety and to preserve the key role of honey bees in the environment, by reducing both direct and indirect beehive contamination [14]. Unfortunately, serious concerns regarding both organic and non-organic honey production are still present. In fact, many persistent environmental organic pollutants (POPs) may contaminate bee matrices [8]. Among the environmental contaminants, in the literature, the presence of polychlorobiphenyls (PCBs), polybromodiphenylethers (PBDEs), organochlorines (OCs), and organophosphates (OPs) in honey samples is well-recognised. In particular, OCs are extremely stable, slightly volatile, lipophilic, and persistent. For these reasons, OCs accumulate and bio-accumulate in foodstuff, representing a matter of concern for the consumer [2]. Organophosphates can induce acute poisoning via food consumption due to their acetylcholinesterase inhibition activity, representing a life-threatening concern [15,16]. This class of pesticides, widely used in agriculture to protect against crop-eating insects and to control *Varroa destructor*, represents a consistent contamination source [17,18]. Due to the common beekeeping practice of recycling old wax combs, OP residues accumulate over time, increasing the potential contamination of the following cycle [19,20]. Due to the high lipophilicity, OPs accumulate, particularly in beeswax, as reported in studies conducted in Belgium [21], France [22], Germany [23], Switzerland [12], Italy [24,25,26], and Spain [3,27,28]. Significant OP levels have also been found in apiaries outside of Europe, such as in North America [29,30,31,32] and South America [33]. Among pesticides, glyphosate (GLY) is the most widely used herbicide in the world [34] and represents a chemical model for estimation of the potential toxic effects on non-target organisms. Although this herbicide exhibits a low toxicity to adult honey bees [35], GLY has recently been associated with sub-lethal health issues in bees due to its chronic accumulation in the hive [36], representing a potential risk for food safety. Although other pesticides have been detected in honey bee products, such as royal jelly and wax combs, there is a lack of information on GLY incidence in these matrices [4]. Due to the analytical difficulties in detecting GLY and its metabolite—aminomethylphosphonic acid (AMPA)—by conventional methods due to their physical and chemical properties [37], it is important to develop reliable analytical methods to monitor their presence, fate, and levels in bee hive product samples, as reported in our previous work [7]. Honey is one of the matrices of animal origin monitored in the Italian National Residue Monitoring Plan (NRMP) concerning veterinary drugs, forbidden and unauthorized substances, and environmental contaminants, such as pesticides; its application aims to guarantee honey traceability and safety and preserve public health and apiaries’ ecosystem [38]. In addition, honey could represent a useful indicator for addressing the environmental pollution of geographical areas.

According to the Council Regulation 1804/1999, the use of allopathic chemically-synthesized medicinal products for preventive treatments in organic beekeeping is prohibited and it is also established that plants that can be foraged by bees must be at least 3 km from any source of pollution and from any non-agricultural production sources [14]. At present, few data are available on the multiresidue screening of xenobiotics oriented to assess the relation between the production context and the consequent potential risk of honey contamination with POPs and non-persistent pesticides. In addition, scarce information is available with regards to the monitoring plans of glyphosate and their metabolites in honey. This is particularly important for organic honey production that may be negatively influenced by production area pollution. Considering this scenario and the constant need to collect data regarding the presence of various xenobiotics in different geographical areas, the aim of the present study was to investigate the presence of contaminant residues using different analytical methods in organic honey samples, from different production areas, to confirm their incidence and possible impact on the food safety traits of organic production.

## 2. Materials and Methods

### 2.1. Honey Sample Collection

**Figure 1:**
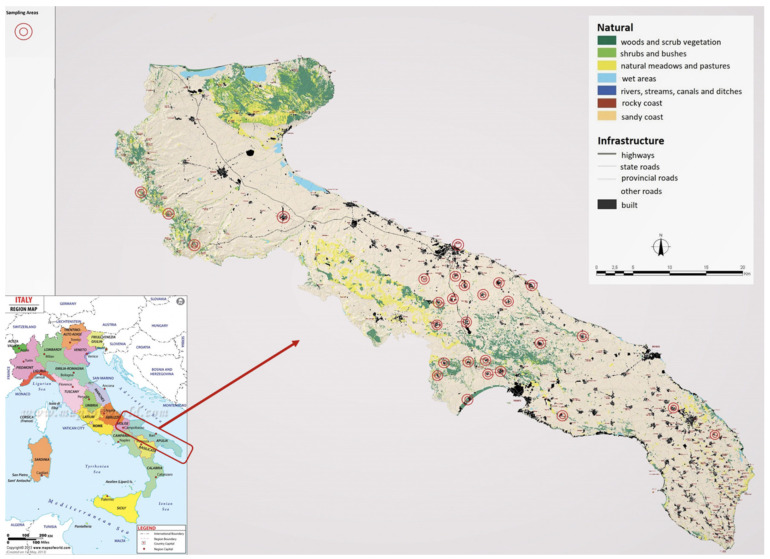
Geographical location distribution of the sampled areas [9].

Ninety-eight honey samples were collected during 2019 and 2020 from different areas in southern Italy (Apulia region), as detailed in Table 1. The distribution of sampled areas according to their geographical location is illustrated in Figure 1.

### 2.2. Chemicals and Reagents

Glyphosate, glufosinate ammonium, AMPA, and the internal standard N-acetyl-d3-glufosinate were obtained from Merck (Darmstadt, Germany). The PCB congener mix containing PCB 28, PCB 52, PCB 101, PCB 138, PCB 153, PCB 180, and PCB 209 as an internal standard (IS), as well as the PBDE mixture made up of PBDE 28, PBDE 33, PBDE 47, PBDE 99, PBDE 100, PBDE 153, PBDE 154, and 3-fluoro-2,2,4,4,6-pentabromodiphenyl ether (FBDE) as IS, were bought from AccuStandard (New Haven, USA). The OC mix, composed of α-HCH, β-BHC, lindane, hexachlorobenzene, heptachlor, heptachlor epoxide, aldrin, dieldrin, endrin, endrin aldehyde, endosulphan I, endosulphan II, endosulphan sulphate, trans chlordane, 4,4′-DDE, 4,4′-DDT, 2,4′-DDT, 4,4′-DDD, and methoxychlor, was obtained from Restek (Bellefonte, PA, USA). The OP mix including anziphos methyl, boscalid, bupiramate, captan, chlorantraniliprol, chlorpyriphos, coumaphos, diazinon, disulphoton, ethoprophos, fenchlorphos, fenthion, fluazinam, iprodion, methyl paration, mevinphos, penconazol, phorate, protiofos, pyraclostrobin sulprofos, quinoxyfen, spirodiclofen, tetrachlorpirophos, tribuphos, trifloxystrobin, and 4-nonylphenol (IS for OCs and OPs) was sourced from Merck (Darmstadt, Germany). Florisil (100–200 96 mesh) was obtained from Promochem (Wesel, Germany). All of the solvents of special grade for pesticide residue analysis (Pestanal) were purchased from Merck. Formic acid (98–100%) was also sourced from Merck (Darmstadt, Germany).

### 2.3. Analysis of Pesticides and POPs

#### 2.3.1. Extraction and Clean-Up

The extraction of POPs was performed according to Chiesa et al. [13], by pressurized liquid extraction with an ASE 350 Accelerated Solvent Extractor (Thermo-Fisher Scientific, Waltham, MA, USA). Briefly, 2 g of honey sample was homogenized with an equal weight of Diatomaceous earth and sodium sulphate and transferred into the extraction cell. Then, 1 mL of isooctane solution containing the three ISs was added and the remaining empty part of the cell was filled with Diatomaceous earth. The cells were packed with a cellulose filter at the bottom, followed by Florisil (5 g). The extraction solvent was a mixture of hexane/ethyl acetate (4:1, *v*/*v*). Organic extracts were then collected and treated with sodium sulphate to remove any possible humidity trace. Finally, the extract was dried in a centrifugal evaporator at 30 °C and dissolved in 200 μL of isooctane.

#### 2.3.2. GC-MS/MS Detection

Pesticides and POPs in honey samples were analysed by triple quadrupole mass spectrometry in electronic impact (EI) mode. A GC Trace 1310 chromatograph through a Rt-5MS Crossbond-5% diphenyl 95% dimethylpolysiloxane fused-silica capillary column (35 m × 0.25 mm, 0.25 μm film thickness, Restek, Bellefonte, PA, USA), coupled to a TSQ8000 detector (Thermo Fisher Scientific, Palo Alto, CA, USA), was used to confirm and quantify residues. The oven temperature program and all of the set mass parameters were described in Chiesa et al. [13]. The Xcalibur^TM^ and Trace Finder 3.0 were the processing and instrument control software programs used.

#### 2.3.3. Extraction of Glyphosate, Glufosinate, and AMPA

The determination of GLY, its metabolite, and glufosinate was conducted according to Chiesa et al. [7]. Briefly, 1g of honey was spiked with the internal standard (100 ng g^−1^) and 3 mL of methanol, followed by the addition of 7 mL of acidified deionized water (1% formic acid). The sample was mixed and then sonicated for 15 min. After centrifugation, 1 mL of the supernatant was filtered by a mixed cellulose syringe filter (0.45 μm) directly into a plastic 2 mL vial.

#### 2.3.4. IC-HRMS Orbitrap Analyses of Glyphosate, Glufosinate, and AMPA

The instrumental analyses were performed by a Dionex ICS-5000+ Ionic Chromatography (IC) system (Sunnyvale, CA, USA) coupled to a Thermo Q-Exactive Orbitrap™ (Thermo Scientific, San Jose, CA, USA), equipped with a heated electrospray ionization (HESI) source. For the analyte separation, a Thermo Scientific Dionex IonPac AS19-4 μm (2 × 250 mm, 4 μm particle size) with a Dionex IonPac AG19-4 μm guard column (2 × 50 mm) was used. The gradient, all IC, and HRMS parameters were described in Chiesa et al. [7].

Chromeleon^TM^ (Thermo Fisher Scientific, San Jose, CA, USA) and Xcalibur^TM^ 3.0 (Thermo Fisher Scientific, Waltham, MA) software was used to control the IC and HRMS system, respectively.

### 2.4. Validation Parameters and Quality Control

The methods were already validated according to SANTE/12682/2019 [39] and accurately described in the above-mentioned studies of Chiesa et al. [13] and Chiesa et al. [7]. For the limit of quantification (LOQ) of the methods, we used the lowest validated spiked level meeting the requirements of recovery within the range of 70–120% and a relative standard deviation RSD ≤ 20%, as defined by the European Commission [39]. Recovery of the studied analytes was carried out at a fortification level of 10 ng g^−1^, while the method repeatability (expressed as the coefficient of variation, CV, %) was evaluated by analysing six replicates for each by adding known quantities of analyte standard solution (10 ng g^−1^) to the honey samples.

## 3. Results and Discussion

This study represents the first survey on the presence of different classes of pesticides and POPs in honey from the Apulia region in Italy. This Italian area was selected for honey sample collection to evaluate the differences with previous research conducted in northern Italy, characterized by industrialized and intensive agricultural contexts [2,13,40]. The results regarding the 98 honey samples are presented in Table 2.

The analytical methods reported here were applied to the investigation of 98 honey samples collected from different Apulian areas to detect and link the occurrence of POPs and pesticides in relation to the contamination source, confirming honey as a suitable indicator of environmental pollution. Apulia region apiculture is strongly based on organic production. Therefore, an evaluation of contaminant residues is critical for sustaining and valorizing organic honey, as well as bee-derived products, such as royal jelly and propolis.

**Figure 2:**
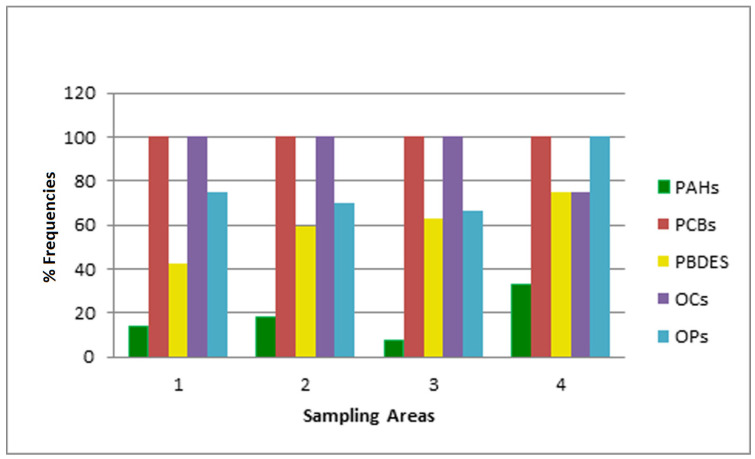
Detection frequencies in the different sampling areas: (1) Intensive orchards; (2) arable lands; (3) areas close to the city without agriculture activities (anthropic sources and traffic); and (4) intensive orchards and arable lands.

The percentage frequencies of detection in the different sampling areas of the different compounds divided into chemical classes are represented in Figure 2.

Regarding POPs, among the four PAHs investigated, traces of benzofluoroanthene were detected in all samples, with higher percentages in honey produced in intensive orchards (14%), arable lands (16%), and mixed intensive orchards/arable lands areas (15%) compared to that produced in anthropized areas (3%). Additionally, chrysene was detected in honey samples from arable lands (8%), anthropized areas (3%), and mixed intensive orchards/arable lands (15%). Another study [40] reported contamination by PAHs in samples of organic honey from various Italian regions, but with higher concentrations of benzofluoroanthene, anthracene, and benzopyrene in high and low anthropized areas. Furthermore, in this case, the presence of PAHs could be due to various sources, both natural (e.g., forest fires) and industrial (e.g., combustion processes at high temperatures), but without specific connections related to the geographical area considered.

Among the six PCBs examined, traces of all congeners at different percentages were detected in almost all of the honey samples, regardless of the sample origin. Specifically, PCB 101 showed the highest (85–100% of samples) and PCB 180 the lowest (38–72% of samples) prevalence. Despite the low concentrations, it is crucial to consider that PCBs were found in all of the sampling areas, confirming that these pollutants are ubiquitous, as reported by other studies [11,13,41]. In fact, the results reported in this study confirm that the PCB concentration in honey, and therefore PCB contamination, is not influenced by the origin of the sample, as confirmed by other studies [2,40,42]. Regarding PBDEs, four congeners (PBDE 99, 100, 153, and 154) were not detected in our samples, according to data on honey collected from other Italian regions reported by Chiesa et al. [13]. Otherwise, traces of the other PBDEs (PBDE 28, 33, and 47) were found at different percentages in samples from all of the geographical areas examined (Table 2). In the literature, few studies concerning the detection of PBDEs in honey are reported (Table S1), with all imputing contamination to direct air transport or also through cross-contamination inside the hive. The results from this study confirmed the ubiquitous presence of many PBDE congeners, underlining the persistence of this class of contaminants in the environment and the consequent possible contamination of both organic and non-organic honey [11,40].

Among pesticides, all of the investigated OCs, with the only exception of β-BHC, which was not detected in this study, were identified in traces in honey samples belonging to all of the geographical areas, as reported in Table 2. The ability of these pesticides to accumulate in the environment and to enter the food chain not only via fatty products, but also via non-fatty products, such as honey, has been previously stated [2]. In particular, some OCs, such as DDT, tend to persist longer than other compounds in the environment, both in an unchanged or metabolized form (DDE and DDD), considering the past use of the parental DDT. The half-life of these pollutants in soil is reported to be over 25 years and strictly related to soil characteristics [43], remaining a threatening issue over time for public health and food chain safety. In this study, different percentages of 2,4′ DDT < 4,4′ DDT < 4,4′ DDD < 4,4′ DDE were detected, in terms of traces, in almost all honey samples tested. Regarding 4,4′ DDD and 4,4′ DDE, the detection percentages were constantly above 55% of samples from each area examined, with the highest percentages being found for DDE in honey from arable lands and anthropized areas (78% and 81%, respectively). These findings, although at higher concentrations, are similar to those reported in other Italian regions, where DDT metabolites were detected more frequently than the parental compound in organic honey samples [13].

Hexachlorobenzene (HCB) was the most frequently identified organochlorine in this study. In fact, traces of this compound were detected in honey samples from all of the geographical areas. As reported in Table 2, intensive orchard areas showed the highest frequency of HCB detection (90%), followed by arable lands and anthropized areas (both 77%) and mixed intensive orchard/arable land areas (69%). This finding is in contrast to previous works regarding Italian honey. Chiesa and colleagues (2016) [13] did not detect this OC in organic honey from Lombardy, Piedmont, and Calabria regions, and Naccari et al. [44] did not report HCB in honey samples from the Sicily region. In another study, only one sample from Emilia Romagna was contaminated with HCB at the concentration of 69.7 ng/g [40]. Hexachlorobenzene can be released as a by-product of chlorination processes such as pesticide production, coal and fuel combustion, and waste incineration [45]. Due to its long atmospheric degradation lifetime and its relatively high vapor pressure and low water solubility, HCB is highly persistent in the environment [46]. In the Apulia region, the occurrence of these sources of contamination, together with the presence of highly polluting industrial sites, could have contributed to the ubiquitous contamination, even at low levels, of the apiaries examined.

The other class of pesticides investigated—OPs—was detected once again in all of the geographical areas examined. Traces of mevinphos, ethopropos, phorate, diazinon, tetrachlorpyrifos, and sulprofos were detected at different percentages (3–7%) in the various sampling sites. Azinphos methyl was detected in traces in 10% of samples from intensive orchard areas and in 4% of samples from arable land areas. Two honey samples from intensive orchard areas showed concentrations of 0.330 and 1.318 ng/g, similar to data reported by Chiesa et al. [13] on organic honey from the Calabria region. The most frequently detected OP in Apulia organic honey was coumaphos. This acaricide, which has been extensively used in recent decades against *Varroa destructor* outbreaks, showed a similar percentage of detection in all of the geographical areas examined. The coumaphos concentration in Apulia organic honey ranged between 0.322 and 2.132 ng/g, which is a result consistent with those reported for Calabria and Trentino organic honey [13]. Although this result could be considered surprising, since the use of allopathic chemically-synthetized medicinal products for preventive bee treatments is prohibited for organic system production, many studies have reported that coumaphos is persistent in wax and can migrate to other bee products, such as honey, in different proportions [17,18]. Moreover, coumaphos is a compound that can also resist the melting temperature of wax, so it is able to accumulate for years, as it is a common beekeeping practice to recycle wax almost continuously in the form of the foundations on which bees construct a complete comb [21]. Consequently, incorrect apiary management by means of *off-label* preventive treatments with acaricide formulations registered for other species and used fraudulently during organic beekeeping operations cannot be excluded either.

No traces of GLY, glufosinate, and AMPA were detected in the samples analysed, demonstrating the food safety of the analysed products and confirming the absence of contamination from agricultural and urban contexts close to the production areas. This result is in accordance with a previous study regarding Italian organic honey marketed in Italy [7]. Our evidence is also echoed in research by El Agraebi et al. [4], where no transfer of GLY from wax to honey was detected. Despite this finding, caution should be taken in the interpretation of the results since the literature confirmed GLY toxicity below regulatory limits [47] and the genotoxicity of AMPA [48].

Moreover, in the study by Berg et al. [49], agricultural lands demonstrated a strong correlation with GLY incidence, with high concentrations when extensive golf courses and/or highways were adjacent to them. In the same study, the authors suggest GLY migration from the site of use into other areas by bees, but in this case, the samples taken directly from 59 bee hives on the Hawaiian island of Kauaʽi were analysed using ELISA techniques.

From the few data reported in the literature, we can also find evidence of GLY’s presence in honey samples, for example, in the study of Pareja et al. [50], where it was detected in 81% of the samples from different origins, with 41% above the MRL. However, none contained AMPA. In the study of Gasparini et al. [51], GLY was detected close to MRL in almost all of the 10 samples obtained from local beekeepers near an agriculture zone. In the study of Chamkasem and Vargo [52], 47% of the samples contained GLY higher than 16 ng g^−1^ (estimated LOQ), while glufosinate and AMPA were not detected in any of the samples.

In general, organic honey produced in the Apulia region displayed contamination by various compounds, although at much lower concentrations than those reported for other Italian regions [2,7,13,40]. Although there does not seem to be a relation in terms of the geographical area considered, generally, the different contaminations detected seem to be mainly linked to the critical points presented by the Apulia region (e.g., highly-polluting industrial sites), together with beekeeping practices that can be considered good, but could still be improved further, especially with regards to organic production. In this study, many POPs and pesticides were detected at a trace level (PCBs, BPDEs, and OCs) or at low concentrations (OPs). While this finding may seem irrelevant from a toxicological point of view, it could actually be a potentially threatening issue for consumer safety, especially for the more fragile categories, such as the elderly and children. In fact, the risk assessment and characterization in terms of the cumulative toxicological effect of low concentrations of multiple xenobiotics have already been addressed. On 29 April 2020, EFSA delivered the first pilot report assessing the cumulative risk from combined exposure to pesticide residues, based on the results of the EU annual monitoring programs for pesticide residues for the years 2014–2016 [53]. Consequently, the monitoring of the safety of honey as a foodstuff (especially from organic production) should be constantly focused on the development of increasingly sensitive analytical methods.

## 4. Conclusions

In this study, the presence of persistent organic pollutants and pesticides was investigated using different analytical methods in organic honey samples from different production areas in the Apulia region. The determination of contaminant residues in the environment and in foods is essential for assessing specific and cumulative human exposure, especially by dietary intake, guaranteeing that it does not exceed acceptable levels for health.

The results of this study show that honey contamination, even at low concentrations (from <LOQ to 2.13 ng g^−1^), is strictly related to highly-polluting industrial site problems of the geographical area, confirming honey bees and beehive matrices as suitable tools for monitoring environmental contamination. With the aim of protecting and increasing the importance of honey production, especially organic production, it would seem mandatory to intensify the safety monitoring of this foodstuff and to keep improving good beekeeping practices, as suggested by the EU framework. Moreover, this approach is critical for developing an integrated strategy to select uncontaminated areas for organic production.

## Figures and Tables

**Table 1:** Characteristics of the different production areas of the 98 collected honey samples.

Sample No.	Area Characteristic in Relation to its Potential Pesticide Sources	Botanical Source
29	Intensive orchards	Acacia, Centaurea, Citrus, *Prunus avium* (Cherry), Eucalyptus, *Prunus dulcis* (Almond), Multifloral, Honeydew
25	Arable lands	Centaurea, Citrus, *Prunus avium* (Cherry), *Coriandrum sativum* (Coriander), Eucalyptus, *Prunus dulcis* (Almond), Multifloral, Honeydew
31	Areas close to the city without agriculture activities (anthropic sources, traffic)	Acacia, Centaurea, Multifloral, Honeydew
13	Intensive orchards and arable lands	Multifloral, *Prunus avium* (Cherry), *Coriandrum sativum* (Coriander), Acacia

**Table 2:** Pesticide residues in 98 honey samples from different geographical areas of the Apulia region (Italy).

Pesticides	Intensive Orchards (*n* = 29)		Arable Lands (*n* = 25)		Areas Close to the City without Agriculture Activities (*n* = 31)		Intensive Orchards and Arable Lands (*n* = 13)		MRLs
	ng g^−1^ ± sd	det. freq.	ng g^−1^ ± sd	det. freq.	ng g^−1^ ± sd	det. freq.	ng g^−1^ ± sd	det. freq.	ng g^−1^ ± sd
**Polycyclic Aromatic Hydrocarbons (PAHs)**									
Chrysene	n.d.	-	<LOQ	2 (8%)	<LOQ	1 (3%)	<LOQ	2 (15%)	-
Antracene	n.d.	-	n.d.	-	n.d.	-	n.d.	-	-
Benzofluoranthene	<LOQ	4 (14%)	<LOQ	4 (16%)	<LOQ	1 (3%)	<LOQ	2 (15%)	-
Benzopyrene	n.d.	-	n.d.	-	n.d.	-	n.d.	-	-
**Polychlorobyphenils (PCBs)**									
PCB 28	<LOQ	23 (79%)	<LOQ	25 (100%)	<LOQ	31 (100%)	<LOQ	10 (77%)	-
PCB 52	<LOQ	24 (83%)	<LOQ	25 (100%)	<LOQ	25 (81%)	<LOQ	9 (69%)	-
PCB 101	<LOQ	28 (97%)	<LOQ	25 (100%)	<LOQ	27 (87%)	<LOQ	11 (85%)	-
PCB 138	<LOQ	25 (86%)	<LOQ	23 (92%)	<LOQ	22 (71%)	<LOQ	10 (77%)	-
PCB 153	<LOQ	27 (93%)	<LOQ	25 (100%)	<LOQ	27 (87%)	<LOQ	12 (92%)	-
PCB 180	<LOQ	15 (52%)	<LOQ	18 (72%)	<LOQ	14 (45%)	<LOQ	5 (38%)	-
**Polybrominated diphenyl ethers (PBDEs)**									
PBDE 33	<LOQ	7 (24%)	<LOQ	5 (20%)	<LOQ	10 (32%)	<LOQ	4 (31%)	-
PBDE 28	<LOQ	6 (21%)	<LOQ	8 (32%)	<LOQ	10 (32%)	<LOQ	6 (46%)	-
PBDE 47	<LOQ	2 (7%)	<LOQ	5 (20%)	<LOQ	3 (10%)	<LOQ	1 (8%)	-
PBDE 99	n.d.	-	n.d.	-	n.d.	-	n.d.	-	-
PBDE 100	n.d.	-	n.d.	-	n.d.	-	n.d.	-	-
PBDE 153	n.d.	-	n.d.	-	n.d.	-	n.d.	-	-
PBDE 154	n.d.	-	n.d.	-	n.d.	-	n.d.	-	
**Organochlorines (OCs)**									
α-BHC	<LOQ	1 (3%)	n.d.	-	<LOQ	1 (3%)	n.d.	-	10
Hexachlorobenzene	<LOQ	26 (90%)	<LOQ	24 (96%)	<LOQ	24 (77%)	<LOQ	9 (69%)	10
β-BHC	n.d.	-	n.d.	-	n.d.	-	n.d.	-	10
ϒ-BHC (Lindane)	n.d.	-	<LOQ	1 (4%)	n.d.	-	n.d.	-	10
Heptachlor	<LOQ	1 (3%)	n.d.	-	<LOQ	1 (3%)	n.d.	-	10
Aldrin	<LOQ	9 (31%)	<LOQ	6 (24%)	<LOQ	5 (16%)	<LOQ	1 (8%)	10
Heptachlor epoxide (isomer B)	n.d.	-	n.d.	-	n.d.	-	n.d.	-	10
trans-Chlordane	<LOQ	1 (3%)	<LOQ	1 (4%)	n.d.	-	n.d.	-	10
Endosulfan I	n.d.	-	<LOQ	1 (4%)	<LOQ	1 (3%)	n.d.	-	10
4,4’-DDE	<LOQ	17 (59%)	<LOQ	21 (84%)	<LOQ	22 (71%)	<LOQ	4 (31%)	50
Endrin	n.d.	-	n.d.	-	n.d.	-	n.d.	-	10
2,4’-DDT	<LOQ	1 (3%)	n.d.	-	<LOQ	1 (3%)	n.d.	-	50
Endosulfan II	<LOQ	2 (7%)	n.d.	-	<LOQ	1 (3%)	n.d.	-	10
4,4’-DDD	<LOQ	17 (59%)	<LOQ	15 (60%)	<LOQ	15 (48%)	<LOQ	7 (54%)	50
4,4’-DDT	<LOQ	5 (17%)	<LOQ	3 (12%)	<LOQ	4 (13%)	<LOQ	1 (8%)	50
Endosulfan sulfate	n.d.	-	n.d.	-	<LOQ	3 (10%)	n.d.	-	10
**Organophosphorus (OPs)**									
Dichlorvos (DDVP)	n.d.	-	n.d.	-	n.d.	-	n.d.	-	-
Mevinphos	n.d.	-	n.d.	-	<LOQ	1 (3%)	n.d.	-	-
Demeton O & S	n.d.	-	n.d.	-	n.d.	-	n.d.	-	10
Ethoprophos	n.d.	-	<LOQ	1 (4%)	n.d.	-	n.d.	-	-
Phorate	n.d.	-	n.d.	-	<LOQ	1 (4%)	n.d.	-	10
Diazinon	<LOQ	2 (7%)	<LOQ	1 (4%)	n.d.	-	n.d.	-	10
Disulfoton	n.d.	-	n.d.	-	n.d.	-	n.d.	-	10
Methyl parathion	n.d.	-	n.d.	-	n.d.	-	n.d.	-	10
Fenchlorphos (Ronnel)	n.d.	-	n.d.	-	n.d.	-	n.d.	-	-
Chlorpyrifos	n.d.	-	n.d.	-	n.d.	-	n.d.	-	50
Fenthion	n.d.	-	n.d.	-	n.d.	-	n.d.	-	10
Trichloronate	n.d.	-	n.d.	-	n.d.	-	n.d.	-	-
Merphos	n.d.	-	n.d.	-	n.d.	-	n.d.	-	-
Tetrachlorvinphos	n.d.	-	<LOQ	1 (4%)	n.d.	-	n.d.	-	-
Prothiofos	n.d.	-	n.d.	-	n.d.	-	n.d.	-	-
Fensulfothion	n.d.	-	n.d.	-	n.d.	-	n.d.	-	-
Sulprofos	<LOQ	1 (3%)	n.d.	-	<LOQ	1 (4%)	n.d.	-	-
Azinphos methyl	<LOQ	3 (10%)	<LOQ	1 (4%)	n.d.	-	n.d.	-	-
	1.32	1 (3%)							
Coumaphos	<LOQ	16 (55%)	<LOQ	18 (72%)	<LOQ	15 (48%)	<LOQ	7 (54%)	10
	0.66±0.2	2 (7%)	1.44±0.3	3 (12%)	0.87±0.2	3 (10%)	1.64±0.4	5 (38%)	

n.d. = not detected (<LOD, limit of detection); LOQ = limit of quantification; sd = standard deviation; det. freq^.^= detection frequency; MRLs = maximum residue limits [38].

## Supplementary Materials

The following are available online at https://www.mdpi.com/2304-8158/9/12/1863/s1, Table S1: Literature data on contaminants and pesticides in honey.
