# Supplementary material for: Pesticides and Environmental Contaminants in Organic Honeys According to Their Different Productive Areas toward Food Safety Protection
Source: Foods. 2020 Dec 14;9(12):1863. doi: 10.3390/foods9121863 (PMC7764946; doi:10.3390/foods9121863)
Supplement: Supplementary file 1 [file foods-09-01863-s001.pdf]

Table S1. Literature data on contaminants and pesticides in honey.

References	Investigated compounds classes	Analytical approach	Selected area traits	Min and Max Concentration Range (Frequency %)
Panseri et al., 2014	Pesticides	GC-MS/MS Ion Trap (IT), Electronic Impact (EI)	Industrialized area (OCPs source)	1.9 - 14.4 ng/g (58.8 - 88%)
			Intensive apple orchard (pesticides utilized in IPM* plan)	3.9 - 18.3 ng/g (25 - 100%)
			Market	3.8 - 8.2 ng/g (27 - 80%)
Chiesa et al., 2016	OCs, OPs, PCBs, PBDEs	GC-MS/MS (triple quadrupole e QqQ)	North Italy (Trentino) - Intensive apple orchard (pesticides utilized in IPMa plan)	0.27 - 13.34 ng/g (5 - 100%)
			South Italy (Calabria) - Intensive citrus orchard (pesticides utilized in IPMa plan)	0.27 - 389.5 ng/g (17 -100%)
			North Italy (Lombardia) - Industrialized area (OCPs, PCBs, PBDEs source)	0.27 - 20.56 ng/g (4 - 100%)
Chiesa et al., 2018	POPs (OCs, OPs, PCBs, PBDEs, PAHs), polar pesticides	LC-HRMS (Orbitrap) and GC-MS/MS (Triple Quadrupole)	High anthropic (HA) impact - North-Italy - Industrialised area (PCBs, OCs source)	118 ng/g Max
			Intensive farming (IF) area - Centre of Italy - Agriculture area (OCs source)	344 ng/g Max
			Farming and husbandry (FH) areas - North-Italy - Livestock and agriculture area (OCs source)	7.98 ng/g Max
			Low anthropic (LA) impact - North-Italy - Free area (no presence of industries or agricultural intensive systems; absence of pesticides)	0.80 - 137 ng/g
			Market	5.93 - 172 ng/g
Amara et al., 2015	Pesticides			0.029 - 410 ng/g (1.5 - 100%)
Rissato et al., 2005	Pesticides	GC-MS-SIM		0.003 - 0.243 mg/kg
Ntirushize et al., 2019	Organochlorine pesticides (OCPs)	GC-ECD	Kabale District, South-Western Uganda	0.11 - 1.53 ng/g (2 - 21%)

Blasco et al., 2003	Pesticides	GC-MS, LC-APCI-MS	Portugal	0.01 - 4.31 mg/kg
			Valencia (Spain)	0.01 - 0.645 mg/kg
				0.02
				0.03
Balayiannis et al., 2008	Pesticides	GC-MS	Citrus groves	0.07 - 9.17 ug/kg
			Natural vegetation (no pesticide); thyme	0.38 - 3.8 ug/kg
			Cotton fields	0.1 - 4.5 ug/kg
			Sunflowers	0.09 - 4.8 ug/kg
Villalba et al., 2019	OCPs, PCBs, PBDEs and Chlorpyrifos	GC-MS	Site 1: farm settled inside a fruit-horticultural belt	10.23 ng/g
			Site 2: agricultural field dedicated to extensive soybean production	10.29 ng/g
			Site 3: field adjacent to urban disposal waste	11.47 ng/g
Mohr et al., 2013	BFRs	GC–QqQ-MS/MS	Brazilian honeys	5.19 pg/g
			Moroccan honeys	4.40 pg/g
			Portuguese honeys	2.24 pg/g
			Spanish honeys	1.77 pg/g
			Slovenian honeys	0.93 pg/g
Roszko et al., 2016	PCB, PBDE and pesticides	GC/MS and LCMS		0.0 - 356.7 pg/g (1.9 - 100%)
Malhat et al., 2014	Pesticides	GC-µECD	Egypt	0.003 - 0.0306 mg/kg
Chauzat et al., 2010	Pesticides	LC-MS/MS, GC-ECD and GC-NPD	France	>LOD - 109.4 µg/Kg (0.9 - 21.8%)
Chaimanee et al., 2019	Pesticides	LC/MS-MS and GC/MS	Agricultural areas in Northern Thailand - Chiang Mai	>LOD - 244 ppb (16.7 - 66.7%)
			Agricultural areas in Northern Thailand - Phayao	4.9 - 58.6 ppb (33.3 - 66.6%)
			Agricultural areas in Northern Thailand - Phrae	>LOD - 106.0 ppb (9.09 - 18.18%)

			Non-agricultural areas in Northern Thailand - Chiang Mai	>LOD - 46.5 ppb (28.6%)
			Non-agricultural areas in Northern Thailand - Lampang	3.1 - 14.0 ppb (33.3 - 50.0%)
			Non-agricultural areas in Northern Thailand - Phayao	5.0 - 80.1 ppb (33.3 - 66.7%)
			A: hedgerow landscape of Loire Atlantique	0.090 - 2.330 µg/kg
			F: hedgerow landscape of Loire Atlantique	0.030 - 0.940 µg/kg
Lambert et al., 2011	PAHs	GC–MS/MS	G: cultivated landscape	0.055 - 0.630 µg/kg
			M: hedgerow landscape of Mayenne	0.120 – 1.230 µg/kg
			IO: Isle of Ouessant of Finistére	0.480 - 5.800 µg/kg
			IY: Isle of Yeu of Vendée	0.060 - 0.730 µg/kg
Karise et al., 2017	Pesticides	HPLC-MS/MS and GC-MS	Estonia	1 - 272 µg/kg
Gaweł et al., 2019	Pesticides	GC–MS/MS and LC–MS/MS	Poland	0.002 - 0.032 mg/kg (1 - 68%)
Bommuraj et al., 2019	Pesticides	GC–MS/MS and LC–MS/MS	Israel	1.4 - 20.8 µg/kg (6.3 - 87.5%)
Raimets et al., 2020	Pesticides	GC-MS and UHPLC-MS/MS	South-eastern Estonia	0.002 - 0.059 mg/kg (3.0 - 27.3%)
			Urban	>LOD - 342 (0 - 71.4 %)
			Suburban/Rural	>LOD - 342 (0 - 52.6 %)
			Developed Open	>LOD - 342 (0 - 87.5 %)
Berg et al., 2018	Glyphosate	ELISA	Agriculture	>LOD - 342 (0 - 90.5 %)
			Forest	>LOD - 342 (0 - 50.2 %)
			Wetland/Riparian	>LOD - 342 (0 - 17.6 %)
			Water	>LOD - 342 (0 - 3.6 %)

Mukherjee et al., 2009	Pesticides	GC-ECD	India	0.005 - 0.22 mg/kg
López et al., 2013	Pesticides	GC-NPD/μECD		>LOD - 0.038 mg/kg
